# Low-frequency sound affects active micromechanics in the human inner ear

**DOI:** 10.1098/rsos.140166

**Published:** 2014-10-01

**Authors:** Kathrin Kugler, Lutz Wiegrebe, Benedikt Grothe, Manfred Kössl, Robert Gürkov, Eike Krause, Markus Drexl

**Affiliations:** 1 German Center for Vertigo and Balance Disorders (IFB), University of Munich, 81377 Munich, Germany; 2 Department of Otorhinolaryngology, Head and Neck Surgery, Grosshadern Medical Centre, University of Munich, 81377 Munich, Germany; 3 Department Biology II, University of Munich, 82152 Martinsried, Germany; 4 Institute for Cell Biology and Neuroscience, Johann Wolfgang Goethe University, 60438 Frankfurt/Main, Germany

**Keywords:** cochlea, low-frequency sound, spontaneous otoacoustic emissions, noise-induced hearing loss

## Abstract

Noise-induced hearing loss is one of the most common auditory pathologies, resulting from overstimulation of the human cochlea, an exquisitely sensitive micromechanical device. At very low frequencies (less than 250 Hz), however, the sensitivity of human hearing, and therefore the perceived loudness is poor. The perceived loudness is mediated by the inner hair cells of the cochlea which are driven very inadequately at low frequencies. To assess the impact of low-frequency (LF) sound, we exploited a by-product of the active amplification of sound outer hair cells (OHCs) perform, so-called spontaneous otoacoustic emissions. These are faint sounds produced by the inner ear that can be used to detect changes of cochlear physiology. We show that a short exposure to perceptually unobtrusive, LF sounds significantly affects OHCs: a 90 s, 80 dB(A) LF sound induced slow, concordant and positively correlated frequency and level oscillations of spontaneous otoacoustic emissions that lasted for about 2 min after LF sound offset. LF sounds, contrary to their unobtrusive perception, strongly stimulate the human cochlea and affect amplification processes in the most sensitive and important frequency range of human hearing.

## 2. Introduction

For decades, low-frequency (LF) sound, i.e. sound with frequencies lower than 250 Hz, has been considered to largely bypass the inner ear even at intense levels, simply because human hearing thresholds for frequencies below 250 Hz are relatively high. Recent evidence from animal models [1] shows that physiological cochlear responses can be elicited with very LF and infrasound, where hearing in most mammals is poor or non-existent. No data for human subjects are available, but, considering the higher sensitivity of humans for LF sounds compared with most mammals, similar results can be expected [1].

Perceptual thresholds essentially reflect the sensitivity of inner hair cells (IHCs), one class of cochlear sensory cells on the basilar membrane, which are functionally coupled to inner ear fluids [2]. IHCs are therefore sensitive to the velocity of sound-driven basilar-membrane movements, which decreases with decreasing frequency of the sound stimulus. Outer hair cells (OHCs), by contrast, are mechanically linked to both the basilar membrane and the overlying tectorial membrane, and are responsible for active cochlear amplification of sound.

OHCs are sensitive to the sound-driven displacement of the basilar membrane [3], which does not decrease with decreasing frequency. Thus, OHCs are more sensitive to LF sound than IHCs. In addition, at LFs, the transfer characteristics of the middle ear [4] and shunting at the helicotrema [5], a small opening connecting scala media and scala vestibuli of the cochlea, attenuate input to both IHCs and OHCs.

While IHCs are contacted by afferent terminals of the auditory nerve and convey acoustic information to the auditory brain, the OHCs' main task is to detect and mechanically amplify sound waves by fast changes of the length of their cell body. This so-called cochlear amplifier [6] actively generates mechanical energy which is fed back into the cochlear travelling wave and ensures the exquisite sensitivity and wide dynamic range of mammalian hearing. Active cochlear amplification leads, as a by-product, to the formation of otoacoustic emissions, sounds generated in the inner ear which can be recorded in the ear canal. In humans, non-invasive recordings of different classes of sound-evoked otoacoustic emissions (EOAEs) allow indirect access to OHC function. While EOAE measurements following LF sound stimulation indicate LF-induced changes in cochlear and especially OHC physiology [7–9], they cannot probe the cochlea in its original state, as the external sound stimulation required already changes cochlear properties. Spontaneous otoacoustic emissions (SOAEs) are narrowband acoustic signals which are spontaneously emitted by the inner ear in the absence of acoustic stimulation and can be recorded in the ear canal with a sensitive microphone. They are a by-product of active biophysical amplification by OHCs in the cochlea. In humans, they persist over years and are relatively stable in both frequency and level under normal physiological conditions [10]. Two main theories about the mechanisms generating SOAEs exist [11,12] and while fundamentally different in their reasoning, they both share active OHCs as necessary elements. Therefore, and because they do not require external stimulation, SOAEs allow for the most direct, non-invasive access to OHC function. A single study [13] reports changes of two SOAEs after LF exposure. Here, we use SOAE measurements for a comprehensive characterization of LF-induced changes of cochlear physiology and active sound amplification. Specifically, we monitored the sound level and frequency of SOAEs before and after the exposure to a 90 s LF sinusoid with 30 Hz and a level of 80 dB(A). Both the sound level and especially the exposure duration used in this study are well below the limits for noise exposures in normal working environments.

## 3. Methods

### 3.1 Subjects

Data were collected from both ears of 21 normal hearing subjects (13 female, eight male; aged between 18 and 28 years, mean age 21 years) for this study. Thirteen subjects (eight female, five male) had participated in a previous experiment and were known to have at least one SOAE in one ear. The remaining eight subjects (five female, three male) had not been screened for SOAEs before. Both ears of all subjects were tested in this study. All subjects had normal hearing thresholds of less than 10 dB HL between 0.25 and 8 kHz. Experiments were conducted in a double-walled, sound-attenuated booth, and the subjects were seated in a comfortable recliner. They were advised to remain still and quiet during the experiment.

The ethics committee of the University Hospital of the Ludwig-Maximilians University Munich, Germany, in agreement with the Code of Ethics of the World Medical Association (Declaration of Helsinki) for experiments involving humans, approved the procedures and all subjects gave their written informed consent. This included a statement that we cannot exclude potential short- and long-term harm to the inner ear caused by the sound levels involved. We also stated that we considered the risk not greater than the one caused by the sound levels one is routinely exposed to in daily life (e.g. with personal sound systems). The sound level of the intense 30 Hz stimulus was 120 dB SPL, corresponding to an A-weighted level of 80 dB. The accumulated daily LF sound exposure was monitored and controlled to be well within the daily exposure limit for normal working conditions in Germany.

### 3.2 Signal generation and data acquisition

An ER-10C DPOAE probe system (Etymotic Research Inc., Elk Grove Village, IL) was used for recording of SOAEs. The LF tone (30 Hz sine wave, 120 dB SPL, 90 s, including 0.1 s raised-cosine ramps) was supplied by a separate loudspeaker (NSW1-205-8A, Aura Sound Inc., Santa Fe Springs, CA). This loudspeaker was connected to a 50 cm long polyethylene tube (inner diameter 1 mm), the tip of which was fed through the foam ear tip of the ER-10C DPOAE probe, so that it faced the tympanic membrane. The loudspeaker was driven by a RB-960BX power amplifier (Rotel, Worthing, UK).

Signal generation and data acquisition was carried out with a RME Fireface UC 24-bit external sound card (RME, Audio AG, Haimhausen, Germany), operated at a sampling rate of 44.1 kHz. The recorded signal was amplified 30 dB by the preamplifier of the external soundcard. Scripts written in Matlab 7.5 (MathWorks, Natick, MA) and run on an ASUS G60 VX laptop (ASUSTeK Computer Inc., Taipei, Taiwan) controlled the external sound card. The SoundMexPro application (HörTech, Oldenburg, Germany) was employed to use low-latency multi-channel ASIO interfacing in the Matlab environment. The entire recording pathway was checked in an artificial ear (B&K 4157, Brüel & Kjær Sound & Vibration Measurement A/S, Denmark). No artefacts exceeding the noise floor of the system could be detected.

A probe-fit-check procedure preceded and concluded each measurement by presenting a band-stop noise consisting of a low- and a high-frequency band and analysing the ear response using a Fourier transform (FT) analysis. If the probe-fit-check procedure at the end of a trial indicated that the probe position had changed, the trial was rejected and repeated.

For calibration of the LF tone, the amplitude response of the probe microphone was compared with the amplitude response of the measuring microphone of an artificial ear (B&K 4157, Brüel & Kjær Sound & Vibration Measurement A/S) and was corrected accordingly. The level of the first harmonic of the LF tone was at least 50 dB lower than the LF tone level. The level of the LF stimulus was monitored continuously during presentation.

All analyses, statistics and visualizations were carried out with scripts written in Matlab 7.5 (MathWorks).

The level and frequency of SOAEs was followed as a function of time: in the control condition, SOAE level and frequency was recorded for 60 s (14 trials) or 120 s (23 trials) without preceding stimulation. In the LF exposure condition, SOAE level and frequency was recorded for 300 s (11 trials) or 420 s (39 trials) following the LF stimulation.

For each subject and ear, at least one control experiment without LF stimulation was conducted.

### 3.3 Analysis

The recorded time domain signal was divided into consecutive segments of 5 s duration. Spectral averaging was applied to each segment to extract the frequency and level of SOAEs. Specifically, Matlab's *pwelch* function was used with a sliding 22 050 points Hann window (resulting in frequency resolution of 2 Hz) with 25% overlap and a zero-padded FFT size of 44 100 points. SOAEs within a frequency range of 0.5–10 kHz were detected automatically by a custom-written Matlab script: SOAEs were only classified as valid when the power of the SOAE candidate (identified by finding local maxima in the frequency spectrum with a minimum level of −15 dB SPL and a spacing of at least 10 Hz) was significantly higher (*F*-test, critical value: 5.39, see Dobie & Wilson [14]) than the power of the surrounding noise floor. The power of the noise floor was calculated by averaging the magnitudes in two frequency bands (eight spectral lines wide, respectively) surrounding the SOAE candidate, each with a 10 Hz spacing from the SOAE candidate frequency.

The level and frequency of SOAEs classified as valid were then analysed over the full recording length. The maximum frequency and level within a range of ±30 Hz around the identified SOAE peak spectral line were extracted for each time window resulting in a timeseries of SOAE frequency and level with a temporal resolution of 5 s. Values from noisy segments or where the signal-to-noise-ratio was too low (as assessed by the *F*-test, see above) were rejected.

For an estimate of the SOAE change duration, the time course of the SOAE levels was fitted with an underdamped, sinusoidal oscillation. A change-detection algorithm [15] was employed to test whether the observed level and frequency changes of the SOAE timeseries were randomly occurring. For this, the cumulative sum of the SOAE level data points from which the mean of the full timeseries was subtracted, was calculated. Then, the difference between the maximum and the minimum of the cumulative sum timeseries was calculated. A bootstrap analysis (1000 samples) was used to randomly reorder the SOAE timeseries, and the analysis described before was repeated for each of the reordered samples. The confidence level was then determined by calculating the percentage of 1000 bootstrap samples where the difference between the maximum and minimum of the bootstrapped cumulative timeseries was smaller than in the original timeseries. We considered changes in the original timeseries significant when the confidence level was equal to or larger than 99%.

The descriptive statistics for all analysed parameters are given as median (first quartile, third quartile), respectively.

## 4. Results

Without LF sound exposure, 80 SOAEs could be recorded from 16 of the 21 tested young, normal-hearing subjects. The SOAE sound levels recorded in the control condition was 0.6 dB SPL (median, first quartile: −4.5 dB SPL, third quartile: 4.0 dB SPL) with a signal-to-noise ratio of 16.6 dB (11.6, 23.5 dB).

**Figure 1.**
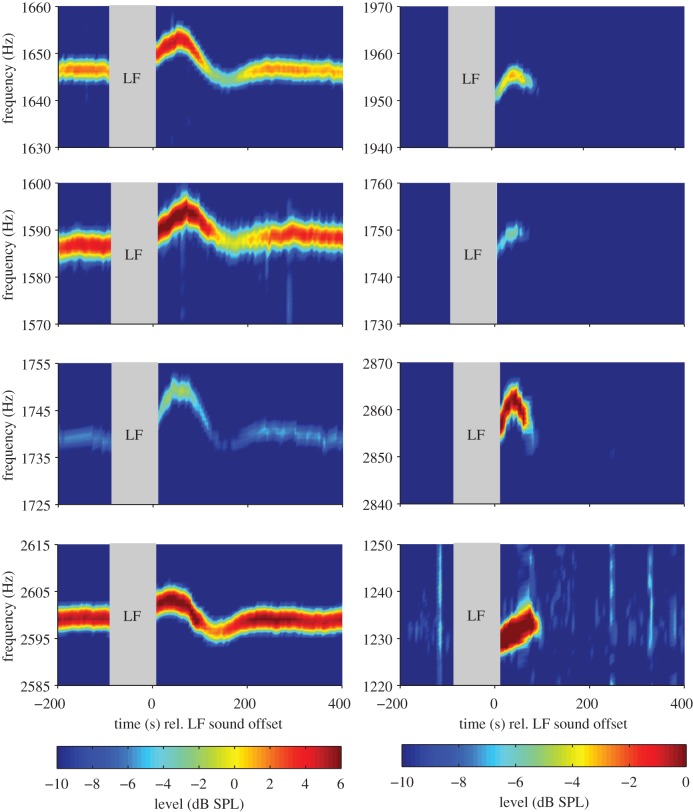
Left column: representative pre- and post LF sound exposure examples of pre-existing SOAE level and frequency changes as a function of time from four different subjects. The grey bar indicates the presentation of the LF stimulus (30 Hz, 80 dB(A), 90 s). The timescale is centred around the LF sound offset at 0 s. Right column: same as in the left column, but for new SOAEs which only appeared for a short period after LF exposure.

**Figure 2.**
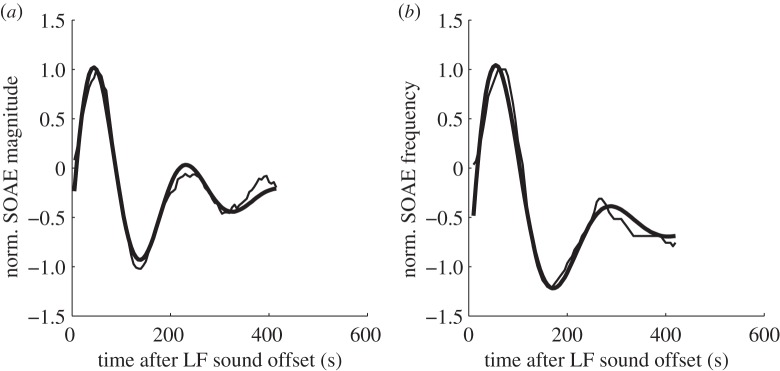
Representative examples of the fits (bold lines) to the normalized changes (thin line, re. maximum level and frequency, respectively) of (*a*) pre-existing SOAE level and (*b*) frequency, which followed an underdamped, sinusoidal oscillation.

After LF sound stimulation, 56 of these 80 SOAEs increased in both sound level and frequency. This increase was followed by a decrease of both level and frequency relative to pre-exposure (figure 1, left column). In 10 of the 80 pre-existing SOAEs from four subjects, we observed an inverted pattern with initial level and frequency decrease with minima about 1 min after the LF exposure, followed by a level and frequency increase after LF sound stimulation. This indicates that in these few cases, the LF-induced changes started earlier within the LF exposure and are thus seen in the falling phase after LF sound offset, similar to what has been reported by Kemp & Brill [9]. All these SOAEs were classified as permanent and bouncing when they fulfilled the following three criteria: they were significantly above the noise floor before the LF sound exposure, level- and frequency changed significantly (confidence level ≥99%), and level- and frequency oscillations could be fitted with an (inverted-phase) underdamped sinusoidal oscillation with *r*^2^>0.8 (see figure 2 for representative examples). *r*^2^ was 0.94 (0.86, 0.97) for the frequency changes and 0.92 (0.87, 0.96) for the level changes. The period of the fitted sinusoid was 257 s (202, 294 s) for the level time course and 252 s (215, 367 s) for the frequency time course. The time constant of the underdamped sinusoid for level changes was 120 s (76, 157 s) and for frequency changes 94 s (58, 141 s). The SOAE frequency changes occurring during the observation interval, expressed as the difference between maximum and minimum frequency during the change, ranged from 2 to 18 Hz. Frequency changes can also be expressed logarithmically as fraction of an octave, where 100 Cent equal a semitone, so 1200 Cent correspond to an octave, and amounted to 5 Cent (4, 9 Cent) with peak values of 25 Cent. Relative to the SOAE frequency in the control condition, the frequency showed initial maximum increases of 4 Cent (3, 7 Cent), followed by maximum decreases of 1 Cent (1, 2 Cent). SOAE level changes were much more pronounced; the absolute difference between maximum and minimum SOAE level after LF presentation was 6.4 dB (4, 9.9 dB), with peak values of 18.7 dB. Maximum SOAE enhancements amounted to 3.3 dB (1.6, 6.6 dB) and SOAE maximum suppressions to 2.7 dB (1, 4.4 dB) relative to the median level of the same SOAE in the recordings without LF stimulation.

**Figure 3.**
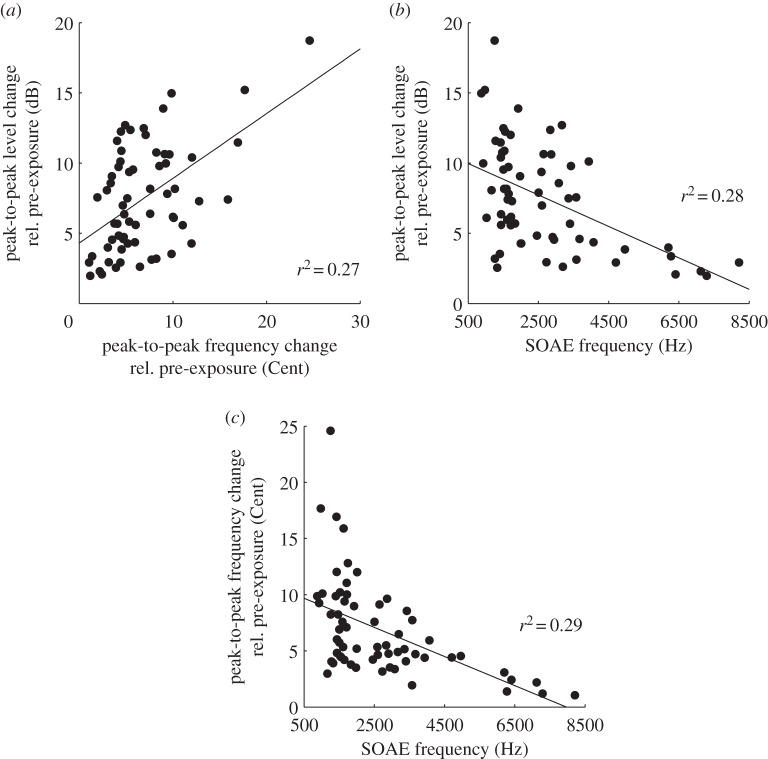
(*a*) Correlation between post-exposure frequency and level changes of bouncing, pre-existing SOAEs. Correlation between the pre-exposure frequency of bouncing, pre-existing SOAEs and (*b*) level and (*c*) frequency changes post-exposure, *p*-values for testing the hypothesis of no correlation against the alternative that there is a non-zero correlation were all smaller than 0.001.

Interestingly, the sign and magnitude of the level- and frequency oscillations of the permanent and bouncing SOAEs were positively correlated, i.e. a large level increase was always accompanied by a large frequency increase, and a large level decrease was always accompanied by a large frequency decrease (figures 1 and 3*a*). Bouncing SOAEs with negative correlations, i.e. a frequency increase with an accompanying level decrease or vice versa, were not observed. Further, the magnitude of both the level and frequency changes depended on the SOAE frequency itself: the lower the SOAE frequency, the stronger were both the level- (figure 3*b*) and frequency changes (figure 3*c*).

**Figure 4.**
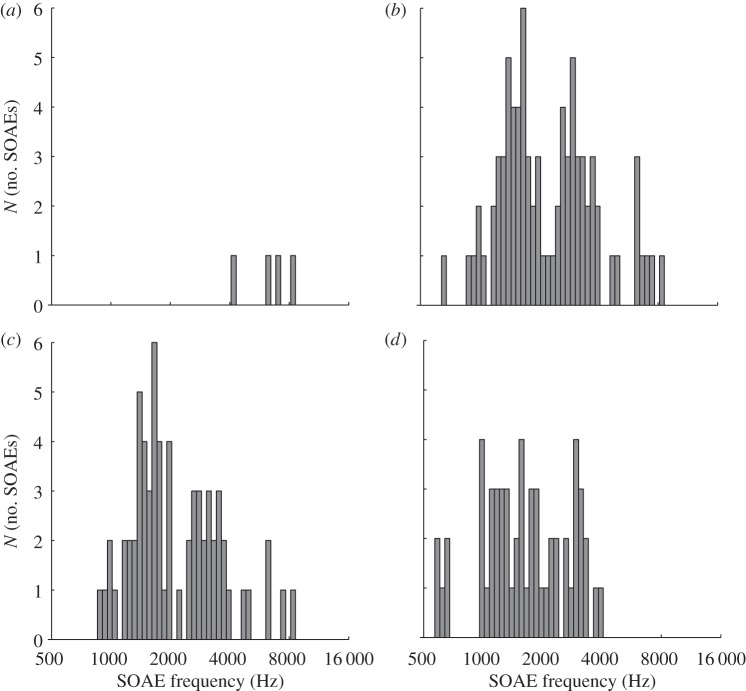
Frequency distribution of pre-existing and new SOAEs. (*a*) Non-bouncing, pre-existing SOAEs post LF-exposure, (*b*) pre-existing SOAEs pre LF-exposure, (*c*) pre-existing SOAEs post LF-exposure, and (*d*) new SOAEs post LF-exposure.

Only four pre-existing SOAEs from four ears of three subjects emerged after the LF exposure with the same, pre-exposure level and frequency (figure 4*a*). These were all relatively high-frequency SOAEs, where the level- and frequency oscillations were also smallest for the bouncing SOAEs. The remaining 10 SOAEs from 10 subjects were not measurable after the LF exposure. Their pre-exposure level was however close to our detection threshold and the post-exposure measurement just failed to reach our rigid significance criterion (see Methods). In addition, in a few cases, pre-existing SOAEs were temporarily suppressed by new, neighbouring SOAEs (see below for a full description of this class) and thus escaped our detection and classification algorithms during that period.

Most interestingly, 17 of the 21 subjects revealed an overall of 56 new SOAEs, which had not been measurable before LF stimulation (figure 1, right column for representative examples). These SOAEs were classified as transient and bouncing when they were not present pre-exposure and the change-detection analysis revealed a significant level change (confidence level ≥99%) in the SOAE time course.

These new SOAEs were characterized by an initial level and frequency increase, qualitatively similar to the pre-existing SOAEs. Comparable to the enhancing half cycle of permanent, bouncing SOAEs, their level and frequency oscillated before they disappeared into the noise floor. The duration of the level and frequency changes was 67.5 s (47.5, 90 s). New SOAEs started to arise within 12.5 s (5, 25 s) after LF sound offset and reached a level maximum at 50 s (35, 62.5 s) after LF offset. The maximum SOAE level was −0.3 dB SPL (−4.1, 4.9 dB SPL) with a signal-to-noise ratio of 13.8 dB (11.9, 17.6 dB). The difference between the new SOAE frequency maximum and minimum was 4 Cent (1, 6 Cent). The time course of level and frequency changes was almost identical and maximum level and frequency changes coincided.

Pre-exposure SOAE frequencies showed a bimodal distribution (figure 4*b*, dip statistic test for unimodality, dip=0.1, *p*=0) with maxima around 1.5 and 3 kHz, in line with previous studies [16,17]. A similar distribution was seen after the presentation of LF sound for permanent, bouncing SOAEs (figure 4*c*, dip statistic test for unimodality, dip=0.1, *p*=0). This is not surprising as the LF sound-induced SOAE frequency changes rarely exceeded 30 Hz and therefore do not affect the overall frequency distribution dramatically. The frequency distribution of new SOAEs, however, differs significantly from this bimodal distribution; (figure 4*d*, dip statistic test for unimodality, dip=0.065, *p*=0.05).

SOAE recordings during the presentation of LF sound were attempted, but because SOAEs can easily be suppressed by external acoustic stimuli, no SOAEs with levels above the noise floor were present, consistent with the findings of Bian & Watts [18], who found that SOAEs were almost completely suppressed with a 75 Hz tone at about 80 dB (A).

## 5. Discussion

The current data show that in humans, active cochlear mechanics, as assessed by SOAE measurements, are significantly affected by LF stimulation. The level and duration of the LF stimulation employed in this study were well below the current limits satisfying national occupational health regulations. The underdamped-oscillatory nature of the observed SOAE level and frequency changes, with a time constant of about 100 s, indicates that a short LF sound exposure of just 90 s significantly changes the cochlear state, so that the recovery process significantly exceeds the exposure duration. This is true despite the fact that the LF sound has a sensation level of only about 60 dB and is not perceived as uncomfortably loud.

LF-induced changes of cochlear mechanics have also been observed with evoked otoacoustic emissions: Drexl *et al.* [7] showed slow oscillations of quadratic distortion product otoacoustic emission after LF exposure with a time course very similar to the present study. Kemp & Brill [9] and Kevanishvili *et al.* [8] recorded click-evoked OAEs after exposure to LF sound with up to 105 dB(A) and also found level changes, albeit not exceeding 1–2 dB, again with a time course very similar to this study.

To the best of our knowledge, this is the first comprehensive study focusing on the effect of LF sound on level and frequency of human SOAEs. Our results are in accordance with Kemp [13], who demonstrated LF-sound-induced changes of frequency and level of two SOAEs and also reported two ‘dormant’ SOAEs which were ‘induced’ by the presentation of LF sound and could only be recorded during the period where existing SOAEs were enhanced. The time course as well as level and frequency changes of the two SOAEs reported by Kemp [13] were similar to our data.

The level and frequency of SOAEs can be influenced by several manipulations. Efferent activity, elicited by the presentation of broadband noise at moderate levels to the contralateral ear, causes frequency increases and accompanying level decreases of SOAEs [19,20]. Likewise, changes of the acoustic impedance difference at the middle ear/inner ear boundary always lead to an SOAE level decrease and accompanying frequency increase [11], regardless of the sign of the impedance change. Impedance changes can be elicited by manipulations of the middle ear pressure [21–23], by postural changes [24,25] or by voluntary [26] or induced [19] contractions of the middle ear muscles. Occasionally, the opposite, i.e. frequency decreases paired with level increases have also been observed during postural changes [25]. In this study, however, we very consistently observed concomitant changes of frequency *and* level with the *same* sign. Also proven theoretically [11], these changes are not compatible with changes at the middle ear/inner ear boundary or changes caused by activity of the efferent system, as these manipulations cause frequency and level changes with opposite signs (see above). In addition, the consequences of the manipulations mentioned above typically outlast the stimulation at most by only a few seconds, whereas the SOAE changes we observed can be detected for more than 100 s after the end of LF stimulation.

Bian & Watts [18] and Bian [27] analysed SOAEs in humans *during* exposure to LF sounds with maximum levels of 50 dB (A), i.e. at least 30 dB fainter than in the current study. Coupled to the phase of the LF tone, the SOAEs showed a periodic level decrease and accompanying frequency increase.

Exposure to loud broadband noise [28] or high doses of salicylate [29] often suppresses SOAEs below the noise floor or causes a prominent reduction in level, accompanied by a frequency decrease. Norton *et al.* [30] presented a range of sinusoids with the lowest stimulus frequency set to 337 Hz, a maximum duration of 300 s and a maximum level of 105 dB SPL to human ears with the intention to observe the recovery of SOAEs after loud sound exposure. Their main finding was a pronounced SOAE-level suppression occurring directly after the offset of the intense stimulation, often with a simultaneous frequency decrease. The SOAE level and frequency quickly recovered to almost pre-exposure levels within about 45–90 s, before a second level- and often frequency minimum around 120 s post-exposure occurred. While the time course of these changes is similar to what we observed, Norton *et al.* [30] consistently observed greatly reduced SOAEs immediately post-exposure, whereas our data show drastically enhanced SOAE levels in most cases.

In summary, it is important to note that only exposure to loud broadband noise and high doses of salicylate, both known to cause auditory thresholds shifts [28,31], have led to positively correlated changes of SOAE levels and frequencies. Activation of the efferent system, the middle ear reflex, head position changes and middle ear pressure changes exclusively cause negatively correlated changes of SOAE frequency and level and are not known to induce threshold shifts. As shown in this study, LF sound causes positively correlated changes of SOAE level and frequency. Contrary to other manipulations causing positively correlated SOAE changes, we observed not only SOAE level suppressions, but also enhancements. Different models have been proposed to explain the generation of SOAEs. The local-oscillator theory (LOT) [12,32] is based on the hypothesis that a self-regulation mechanism balances the viscous damping in the cochlea with electromechanical feedback from the cochlear amplifier. If this feedback mechanism exceeded the cochlear damping, SOAEs would arise as the product of autonomous, self-sustained oscillations of ‘local oscillators’, presumably groups of OHCs locally restricted to a specific place along the cochlea [33]. Another possible mechanism to explain the formation of SOAEs is the global standing wave theory (GST) [34,35]. It states that SOAEs arise from multiple internal reflections of travelling wave energy that produce a stable standing wave between the cochlear boundary and the peak region of a travelling wave. A variant of the GST, the active GST [11,34,36–39] assumes an active amplification process within the cochlea which stabilizes the amplitudes of the standing waves. Both theories, as different as they might be, require the presence of active OHCs, and OHC activity changes should be reflected in the properties of SOAEs in both models.

According to the GST, only a fraction of the possible SOAEs can typically be detected in human cochleae and it has been suggested that several ‘dropouts’ occur [11]. The many new SOAEs seen after the current LF sound exposure can be interpreted as some of these dropouts becoming temporarily detectable. These new SOAEs can be the result of altered cochlear reflection and re-emission related to the gain of the cochlear amplifier in the GST. The fact that new SOAEs preferentially appeared in the distribution minimum of permanent SOAE could indicate that dropouts mainly occur in the corresponding, intermediate frequency range. Because impedance changes at the round window/stapes boundary are not compatible with the changes of SOAE frequency and level we observed, changes most likely involve the mechanical properties of OHCs near the peaks of the forward travelling waves from where backward travelling waves are reflected. These changes are not confined to a point location but rather affect an extended region of the cochlea which is why, during the same period, new SOAEs can arise and existing SOAEs can increase in level. Because the SOAEs according to the GST are standing waves, their frequency is determined by the round trip delay and a delay change will consequently cause a frequency shift. Such a delay change can occur when the stiffness of the OHC is altered. Stiffer OHCs with higher mechanical impedances will cause an increased reflection and re-emission, paired with a phase lead, ultimately causing SOAEs with larger levels and higher frequencies. OHCs with lowered mechanical impedances will consequently lead to weaker SOAEs with lower frequencies. Thus, the observed positive correlation of level- and frequency changes can be explained in the GST model.

Positively correlated SOAE changes can also be interpreted within the characteristics of the LOT: positively correlated decreases of SOAE level and frequency after traumatic noise were successfully modelled in a nonlinear transmission line model where a decrease in gain of the cochlear amplifier resulted in decreasing SOAE levels and frequencies [28]. Thus, according to this model, decreased cochlear amplification should result in decreasing SOAE frequencies and level, and, consequently, increased gain of the cochlear amplifier might result in increased SOAE level and frequency. Nonlinear stiffness oscillators show a dependency of the oscillation frequency on the oscillation magnitude [40]. Accordingly, a change of cochlear gain could result in changed SOAE levels and frequencies, as observed in this study.

To summarize, the very slow, oscillating nature of same-sign SOAE level and frequency changes appears to be a unique feature of exposure to lower frequency sound (i.e. less than 500 Hz). Slow changes of OHC mechanical properties and associated gain of the cochlear amplifier could explain the alterations of SOAE properties we observed. Brief exposures to LF tones have also been shown to induce endolymphatic hydrops [41], possibly altering cochlear mechanics to an extent which can cause, or contribute to, the SOAE changes we observed. It is likely that such induced endolymphatic volume changes share the same origin as the SOAE changes and are a result of LF-induced changes of cochlear homeostasis.

A central element in the control of OHC stiffness (and consequently operating point and gain of the cochlear amplifier) is Ca^2+^ [42]. Hence, possible LF-induced mechanisms leading to Ca^2+^ level changes will be discussed in the following.

Patuzzi [43] suggested that exposure to LF tones induces alterations in the Ca^2+^ homeostasis of OHCs. He argued that only stimulation with LF sound can produce receptor potentials large enough to depolarize the OHC to such an extent that voltage-gated Ca^2+^ channels at the base of the OHCs are opened, triggering Ca^2+^-induced Ca^2+^-release and -uptake, which can become unstable and, as a consequence, can produce oscillation of Ca^2+^ levels.

Intense sound stimulation of the cochlea in the isolated temporal bone preparation has been shown to increase the intracellular Ca^2+^ level of hair and supporting cells [44,45]. This has been considered as one of the signalling pathways involved in noise-induced hearing loss typically accompanied by structural damage to cochlear structures, resulting in temporary or permanent loss of cochlear sensitivity [44,45]. Slow oscillations of Ca^2+^ levels with time constants similar to what we observed have been reported for supporting cells after targeted damage to hair cells [46]. We are proposing that the SOAE changes we observe after LF sound exposure are a reflection of the sound-induced rise of Ca^2+^ levels. Recovery from acoustic overexposure with sounds in the sensitive range of hearing is typically monotonic, does not oscillate and no hypersensitivity can be seen. Acoustic injury consists of a plethora of structural and metabolic changes to the cochlea, with structural damage possibly masking more subtle (and possibly oscillating) changes of cochlear sensitivity caused by the rise of intracellular Ca^2+^ levels. It is feasible that LF sound with the intensities and durations used in this study indeed cause an isolated intracellular Ca^2+^ rise without mechanical damage to cochlear elements.

## 6. Conclusion

The results of this study clearly indicate that there is a pronounced discrepancy between the unobtrusive perception of LF sound, reflected in their low sensation levels and the physiological responses of the cochlea following the LF sound exposure. To the best of our knowledge, perception has been the only measure available in humans to assess inner ear responses to very LF sound, but, as the current data show, severely underestimates cochlear and, especially OHC, sensitivity. Direct quantifications of inner ear active amplification, as measured in this study, are much better suited to assess the risk potential of LF sound.

## Data accessibility

The raw data supporting this study can be accessed under doi:10.6084/m9.figshare.1136017.
